# Editorial: The affective side of Alzheimer's disease (AD): neuropsychiatric symptoms as early sentinel of cognitive decline and pathogenetic factors in disease progression

**DOI:** 10.3389/fpsyt.2023.1197763

**Published:** 2023-07-24

**Authors:** Debora Cutuli, Roberto Coccurello

**Affiliations:** ^1^Department of Psychology, University of Rome La Sapienza, Rome, Italy; ^2^European Center for Brain Research/Santa Lucia Foundation IRCCS, Rome, Italy; ^3^Institute for Complex Systems, National Council of Research, Rome, Italy

**Keywords:** Alzheimer's disease, neuropsychiatric symptoms (NPS), dopamine, ventral tegmental area (VTA), orexin system, risk of dementia

What could we learn from the contributions that have been accepted to be enclosed in this Research Topic focused on the impact of neuropsychiatric symptoms (NPSs) in Alzheimer's disease? Interestingly, we received heterogeneous contributions including quite different aspects of the “affective side” of AD. First, we have learned that depression in AD is not a unitary NPS, which is not equally distributed among AD patients. In the study by O'Bryant et al. “*Depression is differentially related to cognitive and biomarker outcomes among Mexican Americans*”, we found an interesting examination of the higher susceptibility shown by the US Hispanic population, and, in particular, of Mexican Americans, to develop AD in the next 30 years. This is relevant in the context of NPSs because of the increased incidence of AD-associated dementia in the Hispanic and Latino populations (1). O'Bryant et al. also remind us that epidemiological data about the increased risk of dementia in Mexican Americans have been gathered despite the lower rates of positivity to beta-amyloid aggregation. Moreover, it is well-accepted that depression is an affective symptom whose occurrence can predict, with an elevated degree of reliability, the risk of developing AD (2); however, there is a paucity of data concerning the association between depressive symptoms and plasma biomarkers. By comparing a large cohort of Mexican Americans and non-Hispanic subjects, the authors showed that only in the former group was it possible to identify an association between depression score and cognitive decline in non-*APOE*ε4 carriers as well as only between depression and AD biomarkers (e.g., Aβ40, Aβ42, and total tau). As corroborated by plasma biomarkers, the interesting piece of information added to the literature raises the hypothesis that a large part of Mexican Americans can develop a cognitive decline in which depressive symptoms may have a mechanistic role. Despite the limitations intrinsic to cross-sectional studies and the lack of longitudinal analysis, this piece of information well supports the notion that, among NPSs, depression can be highly predictive of AD risk, especially among populations showing the largest increase of diagnosed cases.

Two studies included in this RT deal with separate, yet potentially, overlapping issues. Indeed, the perspective article authored by Krashia et al. discusses the potential mechanistic role of the midbrain ventral tegmental area (VTA) in both AD progression and AD-associated NPSs. The authors point toward the idea, grounded on previous data (3), that early dysfunction/loss of VTA dopaminergic (DAergic) neurons and innervation/output of mesocorticolimbic DA projections toward the prefrontal cortex, nucleus accumbens, and hippocampus might be a key physiopathological AD hallmark contributing to NPSs. In parallel, Bergamini, Coloma, et al. discussed the role of the brain orexin (Ox) signaling system in light of the possible contribution of this neuropeptidergic pathway in the expression of AD-associated NPSs. The authors discussed the role of the Ox system in arousal, aggressive behavior, anxiety, reward processing, and apathy. Apathy and depression are very common in patients with mild cognitive impairment and AD (4), in which a lack of both motivation and feelings of sadness, hopelessness, and incapacity to experience pleasure in everyday activities can coexist. Interestingly, reward processing is blunted by the antagonism of Ox signaling, while the activation of the Ox neurons in the lateral hypothalamus (LH) produces a direct stimulatory effect onto VTA DAergic neurons and the potentiation of mesolimbic DA neurotransmission within the nucleus accumbens (5). LH Ox signaling to the VTA potentiates the evoked mesolimbic DA output-induced place preference and motivation to a food-associated reward-seeking behavior. The implication of the LH Ox-VTA DA circuit for the association between context and rewards can be critical for the study of NPSs and, in particular, for the management of apathy/depression in AD. Considering the role of Ox signaling in memory, stress, and sleep regulation [Bergamini, Coloma, et al.; (6)], the implication of a dysfunctional Ox system in AD (6) should be further investigated within the context of the reduced VTA DAergic input to the prefrontal cortex and ventral striatum (i.e., nucleus accumbens) during the progression of AD (Krashia et al.). Finally, one more point to mention is the contribution by Bergamini, Massinet, et al. in a previous study in which a mouse model of accelerated senescence (i.e., SAMP8 mouse model) has been evaluated for the expression of aggressive behavior in relation to aging and the use of antipsychotic therapy. The study highlights the importance of using mice models of accelerated aging, allowing us to investigate both the impact of age-associated cognitive decline and the incidence of NPSs with the aim of deciphering novel mechanistic links and identifying new potential treatment options.

## Author contributions

RC wrote the paper. DC critically revised the manuscript. All authors contributed to the article and approved the submitted version.
